# Effects of controlled doses of Oxyelite Pro on physical performance in rats

**DOI:** 10.1186/s12986-016-0152-4

**Published:** 2016-12-06

**Authors:** Paulo Vinicios Camuzi Zovico, Victor Magalhães Curty, Marcos André Soares Leal, Eduardo Frizzera Meira, Daniel Ventura Dias, Lívia Carla de Melo Rodrigues, Silvana dos Santos Meyrelles, Edilamar Menezes De Oliveira, Paula Frizera Vassallo, Valério Garrone Barauna

**Affiliations:** 1 Department of Physiological Sciences, University of Espírito Santo, Av. Marechal Campos, 1468 – Maruípe, Vitória, 29043-900 Brazil; 2 UNIPAMPA, Federal University of Pampa, Uruguaiana, Brazil; 3 School of Physical Education and Sport, University of Sao Paulo, Sao Paulo, Brazil

**Keywords:** Dietary supplements, Performance, Liver, Oxidative stress, Mitochondrial biogenesis

## Abstract

**Background:**

OxyElite Pro (OEP) is a dietary supplement to increase metabolism which contains as key stimulant the ingredient 1,3-dimethylamylamine (DMAA). Serious adverse effects have been reported after OEP consumption however, these effects are related to poisoning or overdose. To our knowledge, no one studied the effects of OEP at controlled doses. Thus, the aim of this study was to evaluate acute and chronic OEP affects, at controlled doses in Wistar rats, on physical performance, metabolic parameters, liver injury markers and oxidative stress markers and mitochondrial biogenesis in skeletal muscle.

**Methods:**

Rats were divided in control, 4.3 mg OEP/kg, 12.9 mg OEP/kg and 25.8 mg OEP/kg. All groups were submitted to supplementation with OEP for 4 weeks and the experimental protocols were performed 30 min after the first OEP administration (acute response) and 30 min after the last OEP administration at the end of the forth week (chronic response).

**Results:**

Running distance and running time increased after acute administration of 12.9 mg OEP/kg (2.6-fold) and 25.8 mg OEP/kg (2.8-fold). Since no effect on the exercise tolerance test was observed at the lower OEP dose (4.3 mg OEP/kg), this group was removed from further analyzes. On other hand, running distance and running time decreased after daily supplementation for 4 weeks also in both groups (64% in 12.9 mg OEP/kg and 72% in 25.8 mg OEP/kg). Chronic supplementation at both 12.9 and 25.8 mg OEP/kg decreased TBARS levels in soleus muscle (36 and 31%) and liver (43 and 25%). AOPP was also decreased by both doses in the liver (39 and 45%). Chronic administration of the highest dose, 25.8 mg OEP/kg, was able to reduce mRNA expression of PGC-1α in soleus muscle (25%). No effect was found in other analyses such as spontaneous physical activity, body weight, food and water intake, hepatic toxicity, cardiac oxidative stress and mitochondrial DNA amount.

**Conclusion:**

Maximum and not recommended doses of OEP ingested acutely presented stimulating effect on the ability to exercise. However, its daily consumption for 4 weeks showed antioxidant effects in soleus muscle and liver which may have decreased the PGC-1α mRNA expression on soleus muscle and contributed to the impaired performance in the exercise tolerance test.

## Background

Dietary supplements are a great business worldwide. Since 2004 the supplements industry increased their sales, reaching an approximate value of 61 billion in 2008 only in USA. The sale of dietary supplements continue to increase in popularity, with 80% of adults in the United States buying at least 1 supplement yearly [1]. The number of supplements found on the market increased from 4.000 in 1994 to more than 55.000 products in 2012 [2]. These supplements are sold with the promise of losing weight and increase physical activity performance [3–5]. Most of these dietary supplements contain one or more ingredients capable of stimulating the Central Nervous System (CNS).

OxyElite Pro (OEP) is a dietary supplement produced by the USP Lab (USPLabs, Dallas, TX) and contains a mixture of plant-derived extracts, commercialized in order to aid in weight loss and improve physical performance [5, 6]. The OEP contains a combination of several ingredients that promises to increase metabolism and lipolysis. Among its composition a key ingredient 1,3 dimethylamylamine, also known as germanium, geranamine or DMAA is found [6]. DMAA is a simple aliphatic amine found naturally in geranium flowers (Genus *Geranium*). It is a central nervous system stimulant that induces transient sympathomimetic effects, acting as a norepinephrine reuptake inhibitor and norepinephrine releasing agent [6–8].

DMAA was initially used as a nasal decongestant called Forthane® by the pharmaceutical laboratory Eli Lilly, ending its use in 1983 [9]. Registered as geranamine the DMAA reappeared on the market in 2006 as an ingredient in various dietary supplements [10]. However, several adverse effects began to be associated with the use of products containing DMAA. After these reports, discussions on the origin of DMAA were questioned as to the possibility of this substance be of natural origin or not. Consequently, studies began to investigate geranium plants to identify the presence of DMAA and determine the amount of this substance. In contradiction, some studies found DMAA in geranium plants (funded by USPlabs) [11, 12] and others did not identify the DMAA being of native origin [13, 14]. In addition, the amount of DMAA found in plants was lower than the amount found in dietary supplements.

In April 2013, the Food and Drug Administration (FDA) banned the commercialization of dietary supplements containing DMAA due to the controversy on the amount of DMAA found in plants and into he supplements [4]. However, DMAA has still been found in the general public. DMAA was detected in 25% urine samples collected from portable street urinals in central London [15, 16].

Although some studies have already been performed in humans as described, only 1 study was performed in animal model and only 1 study in cell culture [6, 10], showing the lack of mechanistic studies. Also, studies showing that those supplements are safe and well tolerated were from groups sponsored by its manufacturer, the USP labs [1, 3, 17, 18]. On the other hand, those studies showing serious adverse effects associated with OEP consumption were associated with poisoning or ingestion of a huge or unadvertised amount of the supplement. Thus, the aim of this study was to evaluate the effects of known doses of OEP on physical performance, skeletal muscle oxidative stress and metabolism in animal model.

## Methods

### Experimental groups

Male Wistar Rats (*Rattus Norvegicus Albinus*) 6 weeks-old were obtained from the Federal University of Espirito Santo animal care, Brazil. Rats were kept in groups of five in plastic cages with controlled temperature (22–23 °C), light–dark cycle of 12:12-h, with free access to food and water. All protocols and surgical procedures used were in accordance with the guidelines of the Brazilian College for Animal Experimentation and were approved by the Ethics Committee of the Federal University of Espirito Santo (CEUA-UFES, Protocol 007/2015).

OEP dosages were determined in accordance to doses recommended on the label of the OEP: 1 capsule to 70 kg-adult body weight (i.e. 4.3 mg/kg). Animals were randomly separated into four groups: control group (Vehicle-5% tween20), 4.3 mg OEP/kg (equivalent to 1 capsule, the minimum recommended dosage per day), 12.9 mg OEP/kg (equivalent to 3 capsules, the maximum recommended dosage per day) and 25.8 mg OEP/kg (equivalent to 6 capsules, overdose and not recommended). OEP was diluted in 5% Tween20 and orally administrated by gavage.

Rats were daily supplemented for 4 weeks. Acute data were obtained 30 min after the first OEP administration while chronic data were obtained after 4 weeks supplementation. The same animals were used in both protocols. Since no difference was observed in the exercise capacity after acute 4,3 mg OEP/kg administration, this dose was not administered for 4 weeks.

At the end of the experimental period, rats were anesthetized with an i.p injection of a combination of 100 mg/kg ketamine and 10 mg/kg xylazine, and were euthanized via intravenous (i.v) ketamine injection. Plasma was collected and stored at−80 °C for later analysis. At the end of the protocol, skeletal muscle (soleus and gastrocnemius), heart, liver and adrenal glands samples were surgically removed. The tissues were weighed, washed in a solution of Phosphate Buffered Solution and stored at−80 °C.

### Open field test

Open field test was used to assess spontaneous physical activity as previous published by Rosic et al. [19]. The open field apparatus consisted of a black acrylic enclosed square arena of 43,2 cm × 43,2 cm closed by a wall of 30,5 cm high. At beginning of the test each rat was placed in the center of the arena. Rat movements were recorded by a digital video camera placed centrally above the open field for 5 min and analyzed using AnyMaze software. Spontaneous physical activity was assessed 30 min after the first OEP administration (acute response) and 30 min after the last OEP administration at the end of the forth week protocol (chronic response).

### Exercise Tolerance Test (ETT)

Exercise capacity was determined by graded treadmill exercise test, a method used for detecting exercise intolerance as previous used by our group [20]. Rats were adapted to treadmill exercises for 3 consecutive days with a gradual speed increase (7 m/min, 9 m/min and 13 m/min, without inclination, for 10 min each day). Forty-eight hours after the adaptation period, rats were placed in the exercise streak and allowed to acclimatize for at least 30 min. Exercise began at 7 m/min with no grade and increased by 3 m/min every 3 min thereafter until exhaustion. The ETT was performed following the open field test.

### Food and water intake

Rats were placed in individual metabolic cages (Tecniplast 304) during 48 h for analysis of food and water consumption as previous published by Berger et al. [21]. The first 24 h were used for adaptation and the following 24 h were used to record food and water intake. Metabolic cage was performed 30 min after the first OEP administration (acute response) and 30 min after the last OEP administration at the end of the forth week protocol (chronic response).

### Oxidative stress

The levels of lipid peroxidation were determined using the thiobarbituric-acid reactive substances (TBARS) spectrophotometric assay based on the reaction between malondialdehyde (MDA) and triobarbituric acid (TBA) as previous described by our group Leal et al. [22]. Briefly, the samples were homogeneized with trichoroacetic acid and butylated hydroxytoluene. After being vortexed, the samples were placed in dry bath and then centrifuged. The upper phase was diluted with triobarbituric acid and placed in dry bath for 30 min, and the upper phase was read at 532 nm using a spectrophotometer.

Additionally, the advanced oxidation protein products (AOPP) assay protocol was used to evaluate the oxidation of proteins as previous described [22]. Briefly, samples were diluted with phosphate buffer solution, potassium iodide (KI) and acetic acid, vortexed for 6 min and then measured at 340 nm. Chloramine-T absorbance at 340 nm was used as standard curve (0 to 100 μM). Total protein content was determined by the Bradford method.

### Toxicity

Alanine transaminase (ALT), aspartate transaminase (AST) and γ-glutamyltransferase (GGT) enzymes activities were measured in plasma, using commercially available kits from Bioclin (Brazil), in accordance with the manufactures’ instructions. A standard curve was constructed using stock solution of transaminases and the substrates. All samples of transaminases were measured at 505 nm and GGT was measured at 405 nm.

### mRNA quantification using real-time PCR

The relative gene expression of Peroxisome proliferator-activated receptor coactivator 1 alpha (PGC-1α) was analyzed by real-time PCR. Frozen LV and skeletal muscles samples were homogenized in Trizol and total RNA was isolated according to the manufacturer’s instructions (Invitrogen Life Technologies, Strathclyde, UK). Total RNA concentration and integrity were assessed and real-time PCR was performed. The mRNA expression was assessed by oligonucleotides primers as follows: PGC-1α, 5′-ACC AAA CCC ACAGAGAACAG-3′ and 5′-GGGTCAGAGGAAGAGATAAAGTTG-3′. The expression of cyclophilin A, 5′-AATGCTGGACCAAACACAAA-3′ and 5′-CCT TCTTTCACCTTCCCAAA-3′; was measured as an internal control for sample variation in RT reaction. Quantification of the target genes expression was performed with a SYBRgreen PCR Master Mix (Applied Biosystem, CA, USA). The relative expression of the mRNA was performed by real-time PCR in the ABI PRISM 7700 Sequence Detection System (Applied Biosystem).

### Mitochondrial DNA assay

Mitochondrial to nuclear DNA ratio was used to estimate mitochondrial copy number in skeletal muscle tissue. Skeletal muscles DNA were extracted using Trizol following manufacturer instructions (Invitrogen Life Technologies, Strathclyde, UK). DNA template was amplified by real-time PCR to determine relative mitochondrial and nuclear DNA quantity using SYBRgreen PCR Master Mix (Applied Biosystem, CA, USA). The following primers for ND1 gene (NADH dehydrogenase subunit 1) 5′-TCGGAGCCCTACGAGCCGTT-3′ and 5′-AGGGAGCTCGATTTGTTTCTG-3′; and for nucleus-encoded 18S RNA gene, 5′-TAGTTGGATCTTGGGAGCGGG-3′ and 5′-CCGCGGTCCTATTCCATTATT−3 were used. The expressions of the mitochondrial and nuclear DNAs were performed by real-time PCR in the ABI PRISM 7700 Sequence Detection System (Applied Biosystem).

### Statistical analysis

The values are expressed as means ± SEM. ETT, Open Field and Body weight were analyzed by two-way analysis of variance (ANOVA) for repeated measures followed by Bonferroni’s *post hoc* test. Food and water intake, AST, ALT and GGT, and oxidative stress were analyzed by one-way analysis of variance (ANOVA) followed by Bonferroni’s *post hoc* test. A value of *p* < 0.05 was considered statistically significant. Statistical software SPSS version 22.0 was used for all analysis.

## Results

### Exercise Tolerance Test (ETT)

**Fig. 1:**
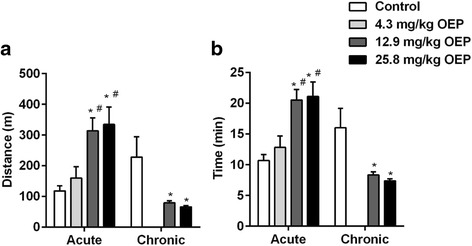
Effect of OEP supplementation on exercise capacity. **a** Total distance, **b** Total time. Data are expressed as mean ± SEM. OEP, Oxyelite Pro. ^*^ *p* < 0.05 vs. Control group; ^#^ *p* < 0.05 vs. 4.3 mg/kg OEP group. Acute, a single OEP administration; Chronic, 4 weeks of daily OEP administration

OEP stimulatory effect on exercise capacity was studied using the ETT. Figure 1 shows ETT after acute and chronic OEP supplementation. Running distance (Control, 118 ± 16; 4.3 mg OEP/kg, 159 ± 37; 12.9 mg OEP/kg, 313 ± 42; 25.8 mg OEP/kg, 334 ± 48, meters, *p* < 0.01, Fig. 1a) and running time (Control, 10.7 ± 0.9; 4.3 mg OEP/kg, 13.9 ± 1.9; 12.9 mg OEP/kg, 20.5 ± 1.7; 25.8 mg OEP/kg, 21.1 ± 2.3, minutes, *p* < 0.01, Fig. 1b) increased only after acute 12.9 mg/kg and 25.8 mg OEP/kg administration. No difference was observed in 4.3 mg OEP/kg and thus this group was not maintained in the chronic protocol.

When rats were daily supplemented for 4 weeks with 12.9 mg/kg or 25.8 mg/kg OEP, running distance (Control, 228 ± 66; 12.9 mg/kg OEP, 79 ± 6; 25.8 mg/kg OEP, 65 ± 4, meters, *p* < 0.01, Fig. 1a) and running time (Control, 16 ± 3.2; 12.9 mg/kg OEP, 8.3 ± 0.5; 25.8 mg/kg OEP, 7.4 ± 0.3, minutes, *p* < 0.01, Fig. 1b) decreased compared to control group.

These data suggest a positive effect of acute OEP supplementation but a negative effect on exercise capacity when OEP is administered continuously for 4 weeks (chronic).

### Spontaneous physical activity

**Fig. 2:**
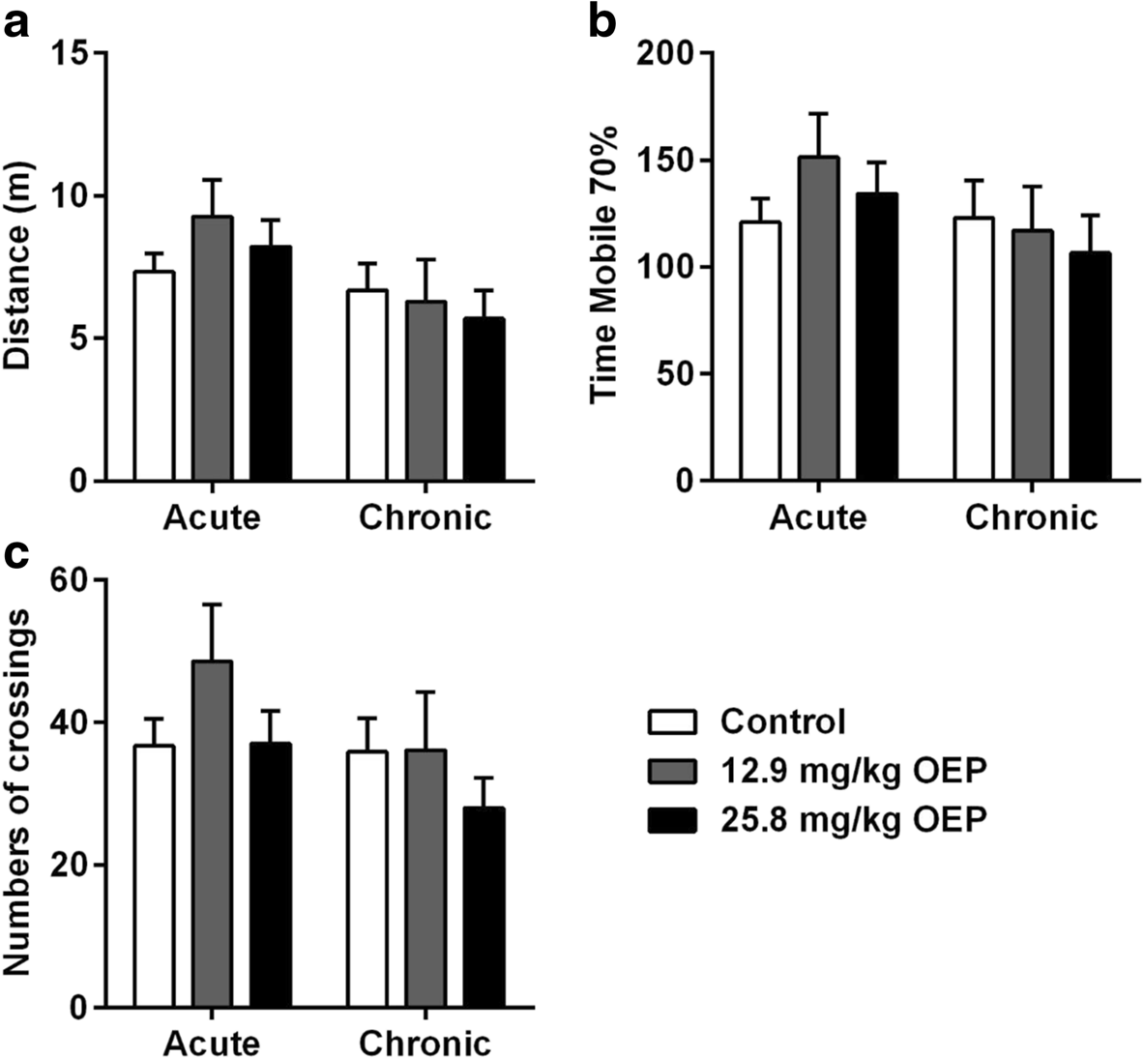
Effect of OEP supplementation on spontaneous locomotor activity. **a** Total distance, **b** Time Mobile 70%, **c** Number of crossings. Data are expressed as mean ± SEM. OEP, Oxyelite Pro. Acute, a single OEP administration; Chronic, 4 weeks of daily OEP administration

Spontaneous physical activity was measured using the open field test (Fig. 2). No significant difference was observed among groups in any of the parameters analyzed: total running distance (Fig. 2a), mobile time (Fig. 2b) and total lines crossed (Fig. 2c). Although not statistically different from Control group, there was a clear tendency to increase spontaneous physical activity after acute OEP supplementation at dose of 12.9 mg/kg OEP while there was tendency to decrease spontaneous physical activity when the higher dose of OEP was supplemented for 4 weeks (Fig. 2, black bars).

### Metabolic parameters and tissue mass

OEP is also a well-known dietary supplement for weight loss. Body weight was analyzed throughout the 4 weeks protocol while water and food intake were analyzed during the first 24 h after the first OEP administration (acute) and during 24 h after the last week of the chronic administration protocol (Table 1).

**Table 1:** Effect of OEP supplementation

Group	Body Weight (BW) (g)		Food intake/BW (mg/g)		Water intake/BW (ml/g)	
	0 WK	4 WK	Acute	Chronic	Acute	Chronic
Control	177 ± 9	318 ± 9	102 ± 5	73 ± 5	0.145 ± 0.009	0.104 ± 0.006
12.9 mg/kg OEP	176 ± 9	314 ± 9	98 ± 5	77 ± 6	0.137 ± 0.005	0.111 ± 0.008
25.8 mg/kg OEP	163 ± 9	298 ± 8	99 ± 5	65 ± 7	0.143 ± 0.010	0.098 ± 0.007

*OEP* Oxyelite Pro. Acute, a single OEP administration; Chronic, 4 weeks of daily OEP Administration. Data are expressed as mean ± SEM

Analysis on body weight, and food and water intake

Body weight was not different among groups at the beginning or at the end of the protocol (Table 1). In agreement, no difference in food or water intake was observed among groups after acute administration or after 4 weeks OEP supplementation measured by the metabolic cage (Table 1). Altogether there is no evidence that either acute or chronic OEP supplementation inhibits appetite or aids to decrease body weight at least in normal feed rats.

Also, at the end of the chronic protocol (4 weeks), gastrocnemius (Control, 4.3 ± 0.13; 12.9 mg/kg OEP, 4.4 ± 0,13; 25.8 mg/kg OEP, 4.6 ± 0.16, g/BW), soleus (Control, 0.41 ± 0.010; 12.9 mg/kg OEP, 0.45 ± 0.012; 25.8 mg/kg OEP, 0.43 ± 0.014, mg/BW), heart tissue (Control, 3.1 ± 0.05; 12.9 mg/kg OEP, 3.2 ± 0.06; 25.8 mg/kg OEP, 3.1 ± 0.06, g/BW), and adrenal glands (Control, 0.081 ± 0.004; 12.9 mg/kg OEP, 0.078 ± 0.005; 25.8 mg/kg OEP, 0.073 ± 0.004, mg/BW) were weighed and no differences among groups were observed.

### Liver injury markers

Cases of acute hepatitis and liver injury were related to the use of OEP, however the amount of OEP ingestion was reported as unknown or at high doses [4, 5, 23]. Other studies with long-term supplementation but with known amount of OEP found no difference in liver injury markers (AST, ALT and GGT) [3, 17, 24].

**Fig. 3:**
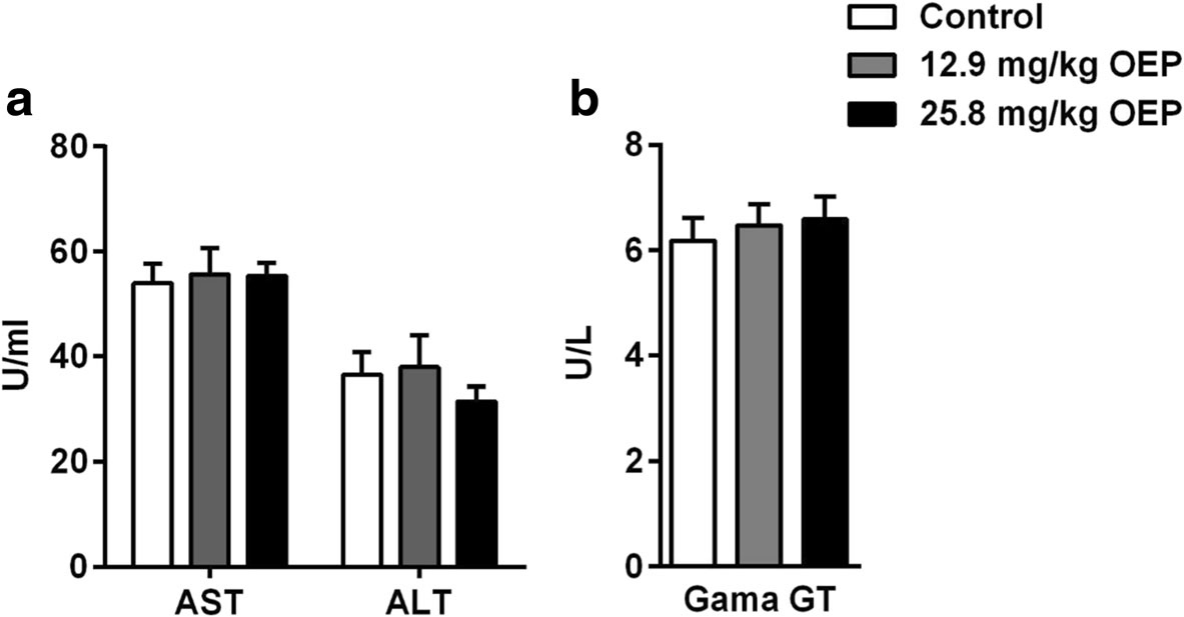
Effect of OEP supplementation on liver injury markers. **a** Aspartate Transaminase, AST; and Alanine Transaminase, ALT; **b** γ-glutamyltransferase, Gama-GT. Data are expressed as mean ± SEM. OEP, Oxyelite Pro

Circulating liver injury markers ASL, ALT and Gama-GT were also measured at the end of the chronic protocol. Fig. 3a shows similar levels of AST (Control, 54 ± 3; 12.9 mg/kg OEP, 55 ± 5; 25.8 mg/kg OEP, 55 ± 2, U/ml) and ALT (Control, 36 ± 4; 12.9 mg/kg OEP, 37 ± 6; 25 mg/kg OEP, 31 ± 2, U/ml) among groups, while Fig. 3b shows no differences in the Gama-GT levels (Control, 6.2 ± 0.4; 12.9 mg/kg OEP, 6.9 ± 0.5; 25.8 mg/kg OEP 6.4 ± 0.4, U/ml).

### Oxidative stress parameters

**Fig. 4:**
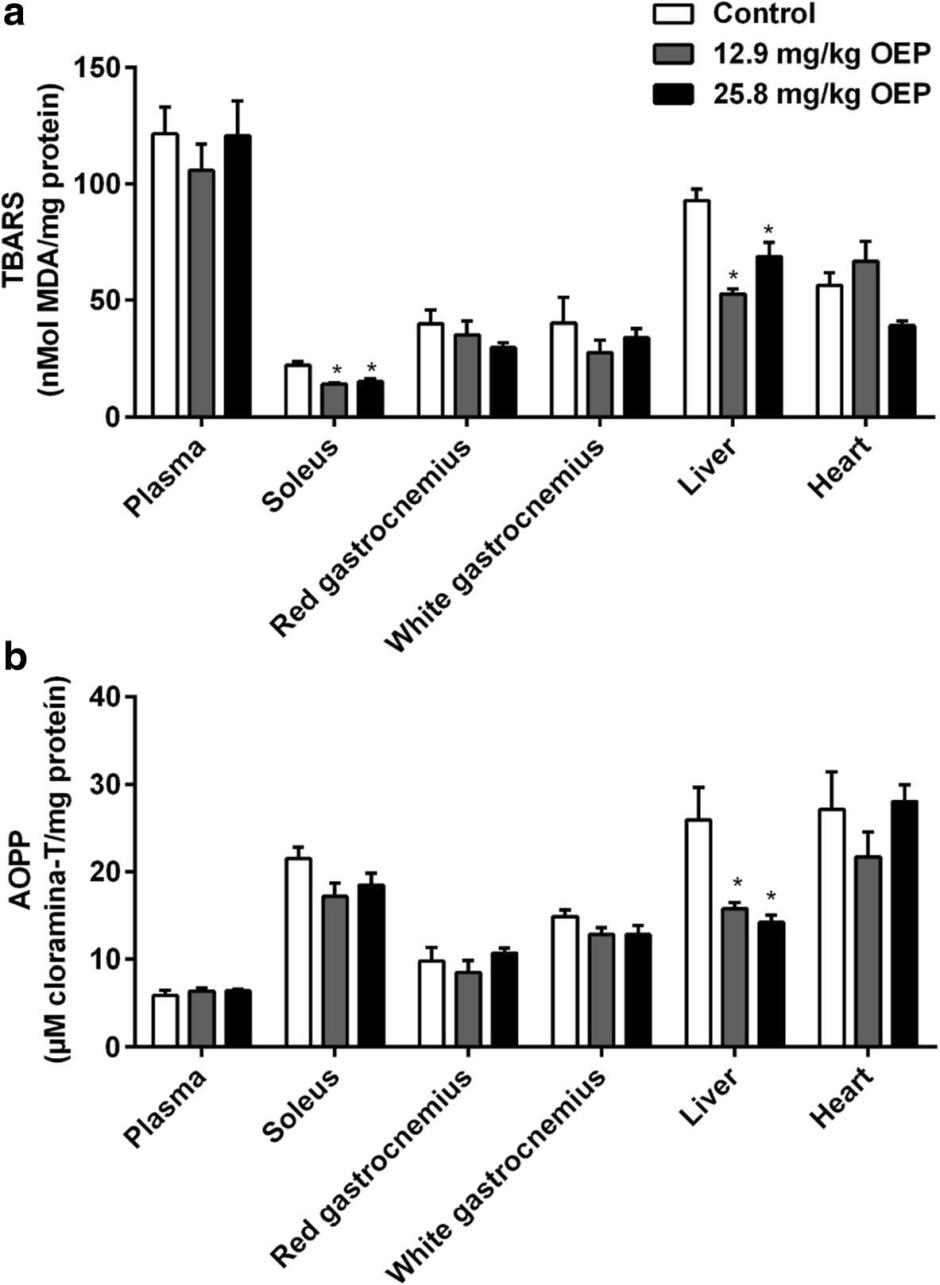
Effect of OEP supplementation on circulating and tissue oxidative stress markers. **a** Lipid peroxidation, **b** Protein oxidation. Data are expressed as mean ± SEM. TBARS, thiobarbituric-acid reactive substances; AOPP, advanced oxidation protein products; ^*^ *p* < 0.05 vs. Control group

Tissue and circulating lipid peroxidation (TBARS, Fig. 4a) and protein oxidation (AOPP, Fig. 4b) were analyzed after 4 weeks OEP supplementation. Plasma, TBARS and AOPP was similar among groups.

Also, red and white gastrocnemius, and heart were similar among groups. However, OEP at both doses decreased lipid peroxidation in soleus muscle (12.9 mg/kg, 36%; 25.8 mg/kg, 31%) while only a tendency to decrease was observed on protein oxidation (12.9 mg/kg, 20%; 25.8 mg/kg, 15%; *p* = 0.11).

Hepatic oxidative stress markers were also decreased by both doses (TBARS; 43% in 12.9 mg/kg OEP and 25% in 25.8 mg/kg OEP; and AOPP; 39% in 12.9 mg/kg OEP and 45% in 25.8 mg/kg OEP).

### Mitochondrial Biogenesis

**Fig. 5:**
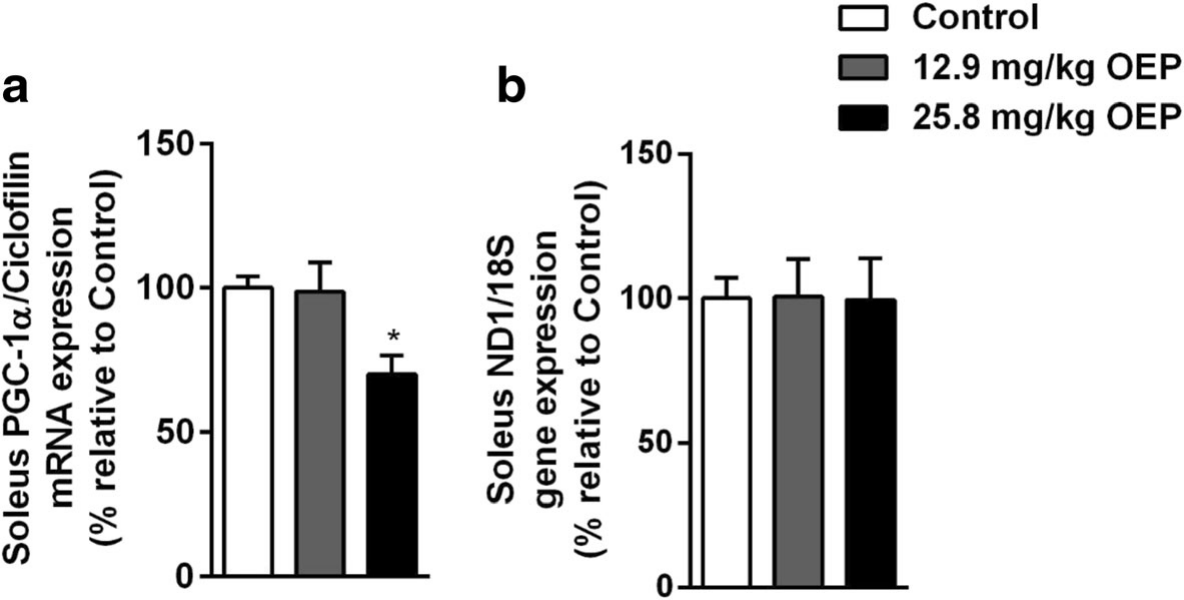
Effect of OEP supplementation on mitochondrial biogenesis markers. **a** PGC-1α mRNA expression, **b** ND1 DNA amount. Data are expressed as mean ± SEM. PGC-1α, peroxisome proliferator–activated receptor coactivator 1-alpha; ND1, NADH dehydrogenase subunit 1; ^*^ *p* < 0.05 vs. Control group

PGC-1α mRNA expression (Fig. 5a) ND1 DNA amount (Fig. 5b) were measured after 4 weeks of OEP supplementation in soleus muscle, which has predominantly oxidative fibers. PGC-1α mRNA expression decreased 25% in 25.8 mg/kg OEP group compared to control group, but no difference was observed in 12.9 mg/kg OEP group (Fig. 5a). ND1 DNA expression, marker of total mitochondria content, was not modified among groups (Fig. 5b).

## Discussion

The findings from our study indicate that a single OEP administration stimulated exercise performance (12.9 and 25.8 mg/kg), while chronic administration for 4 weeks of OEP decreased physical activity. Chronic administration of OEP decreased oxidative stress markers in soleus and liver tissues and decreased PGC-1α mRNA expression. Lastly, OEP supplementation did not change body weight, food and water intake, hepatic injury markers (AST, ALT, Gama-GT) and skeletal muscle total mitochondria amount.

OEP was mainly sold as fat burner and to assist in rapid weight loss. In the present study we found no difference in body weight and food intake of the animals supplemented with OEP. However, data found in the literature are controversial on this subject. The effect of OEP in reducing body weight and appetite was described with 1 or 2 serving doses during 8 weeks by McCarthy et al. [3], and after 2 weeks by Farney et al. [24]. However, it is noteworthy that the subjects of both studies were regularly engaged in physical activity protocols (which may have contributed to the weight loss) and both studies were financed by USP Labs, the OEP manufacturer. Also, our results corroborate with data found by Whitehead et al. [17], that no effect on body weight was observed with supplementation of 1–3 serving OEP during a period of 10 weeks without exercise training.

In our study, OEP showed no significant effect on spontaneous locomotor activity. Although none has yet studied OEP on spontaneous locomotor activity before, Dolan et al. [10] studied the effects of isolated DMAA, one of several ingredients in OEP, on mice spontaneous activity. The authors observed that acute DMAA administration induced depressant effects within 0 to 30 min which lasted for 50 to 70 min, but increased locomotor activity after 120 to 180 min. This apparently disparate data may be attributed to the fact that the dietary supplement OEP has undetermined amount of DMAA and also has caffeine on its formulation, which biased this comparison.

Acute OEP administration at both 12.9 and 25.8 mg/kg doses but not 4.3 mg/kg OEP increased exercise capacity. Bloomer et al. [25] did not observe effect on running time in humans after acute ingestion of DMAA or its combination with caffeine. However, the authors reported that their results may have been biased due to previously daily use of caffeine throughout the protocol informed by the participants. Stimulatory effects of acute ingestion of 6 mg/kg caffeine in rats and 5 mg/kg caffeine in humans has been demonstrated [26]. Also, acute ingestion of 3 mg/kg caffeine or more is capable to improve physical performance and endurance in humans and animals [27]. Due to the lack of data explaining DMAA effects, these findings with caffeine from literature could at least partially explain our results. Each OEP capsule contain 100 mg of caffeine and consequently the dosage used in both studied groups with higher concentration of OEP had at least 3 mg/kg of caffeine, the necessary dose to increase physical activity acute consumption [26, 27]. Thus, we believe that the stimulatory effect observed after acute OEP administration is more likely due to caffeine in the supplement instead of the DMAA amount.

Oppositely to findings from acute administration, 4 weeks of OEP harmed exercise performance. Vaughan et al. [6] observed increase in PGC-1α mRNA and protein expression, and mitochondrial biosynthesis in skeletal muscle cells after 24 and 48 h of OEP incubation. We found, *in vivo*, decreased PGC-1α mRNA expression and unchanged mitochondrial content in the soleus muscle, the main oxidative muscle of rat hind limb. Data from both studies are difficult to compare since they used a cell culture model and doses of OEP that cannot be translated to the recommended doses in humans [6].

The antioxidant effect of OEP observed in our data (reduced levels of MDA and AOPP in soleus muscle and liver) may at least partially explain the decreased exercise performance and PGC1α expression. PGC-1α is a major regulator of energy metabolism and of mitochondrial biogenesis [28]. ROS are essential signaling molecules that contribute to up-regulation for the expression of several genes, including genes related to mitochondriogenesis [29]. There is evidence for an antioxidant role of caffeine. Barcelos et. al. [30] showed that long-term caffeine consumption reduced MDA levels in the liver of exercise-trained rats [30], while Gomez-Cabrera et al. [29] showed that 8 weeks of antioxidant supplementation decreases endurance capacity (running time), mitochondriogenesis and expression of key transcription factors involved in mitochondrial biogenesis. However, there are also studies demonstrating that caffeine increases mitochondrial biogenesis markers as PGC-1α and improves mitochondrial function and biogenesis [31–33]. In addition, we cannot exclude the antioxidant effects of OEP as previous described by McCarthy et al. [3]. The authors observed lower plasmatic MDA levels than placebo after OEP supplementation for 8 weeks with 2 serving doses. Finally, since OEP contains a mixture of ingredients we cannot exclude possible antioxidant effects of other ingredient in the OEP formula.

Serious effects reported in the literature have been associated with use of products containing DMAA which include tachycardia, nausea, vomiting, agitation, tremor, dizziness, headache, confusion, drowsiness, chest pain, palpitations, slurred speech [24, 34]. More serious and life-threatening effects also have been reported, such as acute myocardial infarction [35], cardiac arrest [36], hemorrhage stroke [37–39] and death [16, 40]. In addition, hepatotoxic effects were associated with the use of OEP as: acute hepatitis and liver injury [23]. However, the doses used in these studies are high or unknown and, in many cases, were associated with the use of alcohol, other drugs or strenuous physical exercise. On the other hand, similar to our findings, other studies have observed no difference in the liver injury markers (AST, ALT and GGT) [3, 17, 24, 25]. Differently from the studies that observed hepatotoxicity effects, these studies also used controlled does of OEP recommended in the product label and were not associated with any other drugs.

In summary, our study is the first one to use the recommended dose of OEP in animal model. We did not observe such adverse effects related on literature mainly because those responses are associated with unknown or uncontrolled doses of OEP consumption. Also, we did not observe the weight loss effect of OEP as promised in the product label. On the other hand we observed that acute administration of OEP increased physical performance, while its chronic administration for 4 weeks decreased its performance. We believe that the antioxidant effect, observed by decreased TBARS and MDA, may have contributed to the decreased PGC-1a expression and thus decreased aerobic performance in chronic consumption. Another possible mechanism is the rats tolerance to the supplement compounds, mainly to the caffeine as already demonstrated [41].

One limitation of this study was that our experiments were performed using a non-obese rat model. It would also be interesting to examine the effects of OEP in obese or overweight rat model. Further research is needed to elucidate the effects of OEP on these models.

## Conclusion

Our results suggest that doses maximum and not recommended of OEP ingested acutely presents a stimulating effect on the ability to exercise. However, the use of these doses for 4 weeks showed antioxidant effects in the soleus muscle and liver. Also, consumption of doses greater than the recommended amount may have contributed to suppressed soleus PGC-1α mRNA expression and reduced physical performance of supplemented animals.

## Abbreviations

ALT: Alanine transaminase
AOPP: Advanced oxidation protein products
AST: Aspartate transaminase
CEUA: Ethics committee of the Federal University of Espirito Santo
CNS: Central nervous system
DMAA: 1,3 dimethylamylamine
ETT: Exercise tolarence test
FDA: Food and drug administration
GGT: Gama-glutamyltransferase
KI: Potassium iodide
MDA: Malondialdehyde
ND1: NADH dehydrogenase subunit 1
OEP: OxyElite pro
PGC-1α: Peroxisome proliferator-activated receptor coactivator 1 alpha
TBA: Thiobarbituric acid
TBARS: Thiobarbituric-acid reactives substances
UFES: Federal University of Espirito Santo
